# Exploiting bounded signal flow for graph orientation based on cause-effect pairs

**DOI:** 10.1186/1748-7188-6-21

**Published:** 2011-08-25

**Authors:** Britta Dorn, Falk Hüffner, Dominikus Krüger, Rolf Niedermeier, Johannes Uhlmann

**Affiliations:** 1 Fakultät für Mathematik und Wirtschaftswissenschaften, Universität Ulm, Ulm, Germany; 2 Institut für Softwaretechnik und Theoretische Informatik, TU Berlin, Berlin, Germany; 3 Institut für Theoretische Informatik, Universität Ulm, Ulm, Germany

## Abstract

**Background:**

We consider the following problem: Given an undirected network and a set of sender-receiver pairs, direct all edges such that the maximum number of "signal flows" defined by the pairs can be routed respecting edge directions. This problem has applications in understanding protein interaction based cell regulation mechanisms. Since this problem is NP-hard, research so far concentrated on polynomial-time approximation algorithms and tractable special cases.

**Results:**

We take the viewpoint of parameterized algorithmics and examine several parameters related to the maximum signal flow over vertices or edges. We provide several fixed-parameter tractability results, and in one case a sharp complexity dichotomy between a linear-time solvable case and a slightly more general NP-hard case. We examine the value of these parameters for several real-world network instances.

**Conclusions:**

Several biologically relevant special cases of the NP-hard problem can be solved to optimality. In this way, parameterized analysis yields both deeper insight into the computational complexity and practical solving strategies.

## Background

Current technologies [1] like two-hybrid screening can find protein interactions, leading to protein-protein interaction (PPI) networks, but cannot decide the direction of the interaction. This can be complemented by gene knock-out experiments which constitute a way to determine causal relations in these networks, thus providing additional information on possible directions of information flow in them [2]. Given a list of so-called cause-effect pairs, the challenge consists in deducing an orientation of the PPI network which takes into account the causal relations of as many of these pairs as possible. Medvedovsky et al. [3] formalize this in terms of a graph theoretical problem as follows.

### Problem Formalization

Let *G *= (*V*, *E*) be an undirected graph. An *orientation *$\vec{G}$ of *G *is a directed graph $\vec{G}$ = (*V*, $\vec{E}$) obtained from *G *by replacing every undirected edge {*u*, *v*} ∈ *E *by a directed one, i. e., either by (*u*, *v*) ∈ $\vec{E}$ or by (*v*, *u*) ∈ $\vec{E}$. Let *P *⊆ *V *× *V *be a set of ordered source–target pairs, which we sometimes refer to as "signals". In order to distinguish pairs from edges or arcs, we use the notation [*a*, *b*] ∈ *P *to denote the pair starting in *a *and ending in *b*. We say that a pair [*a*, *b*] ∈ *P *is *satisfied *by a given orientation $\vec{G}$ if there exists a directed path from *a *to *b *in $\vec{G}$. The central problem considered in this work is to find an orientation of a given graph maximizing the number of satisfied pairs. As pointed out by Medvedovsky et al. [3], we can assume that the given graph is a tree: it is clearly optimal to orient the edges of a cycle to form a directed cycle, and hence one can repeatedly contract a cycle to a single vertex, obtaining a tree. Note that this process will always produce the same tree independent of the order of contractions, since two vertices will be merged eventually if and only if they are in the same bridge block, where a bridge block is a connected component of the graph that is obtained by deleting all bridges (edges whose deletion increases the number of connected components). Further, bridge blocks can be found in linear time [4,5]. Thus, formalized as a decision problem, MAXIMUM TREE ORIENTATION is defined as follows.

### Maximum Tree Orientation (MTO)

**Input**: An undirected tree *T*, a set *P *of ordered pairs of vertices of *T*, and an integer *k ≥ *0.

**Question**: Is there an orientation of *T *such that at most *k *pairs in *P *are not satisfied?

We also consider the weighted version, called WEIGHTED MAXIMUM TREE ORIENTATION (W-MTO), where every pair [*a*, *b*] *∈ P *is associated with a rational weight *ω*([*a*, *b*]) ≥ 0, and the goal is to maximize the sum of weights of the satisfied pairs. Throughout this work, *n *denotes the number of vertices in the given MTO instance, if not stated otherwise.

As sketched before, MTO is motivated from the inference of causal relations in biological networks [6,7] such as PPI networks, but it also has applications in the context of communication networks, where several one-way connection request pairs are given. Since each link between two network nodes can only be used in one direction, one has to orient the links in such a way that as many communication requests as possible can be fulfilled.

## Previous Work

MTO was introduced by Medvedovsky et al. [3]; they showed that the problem is NP-complete even when the underlying tree is a star (that is, a diameter-two tree) or a tree with maximum vertex degree three. Moreover, they provided a cubic-time algorithm for MTO restricted to paths. Seeing MTO as the task to maximize the number of satisfied pairs, Medvedovsky et al. also provided polynomial-time approximation algorithms with approximation factor 1*/*4 in the case of stars and *O*(1*/*log *n*) in the case of general *n*-vertex trees. The latter approximation factor was recently improved to *O*(log log *n/*log *n*) by Gamzu et al. [8], who furthermore extended the studies of MTO to "mixed graphs" where some of the edges are already oriented based on causal relations known in advance. Besides these theoretical investigations, Medvedovsky et al. [3] also provided some experimental results based on a yeast PPI network and some synthetic data. Silverbush et al. [9] recently formulated a polynomial-size integer linear program for the generalization of mixed graphs and did some experiments with it. Also recently, Gitter et al. [10] considered graph orientation with the objective of maximizing the weight of all satisfied paths between sources and targets with length at most some constant *k*. They used approximation algorithms to discover pathways in biological networks. In an earlier work, Hakimi et al. [11] studied the special case of MTO where the list of pairs to be satisfied contains *all *possible pairs; they developed a quadratic-time algorithm for this case.

## Our Contributions

We mainly continue and complement previous work on MTO [3,8] by starting a parameterized and multivariate complexity analysis of MTO. That is, we try to better understand the border between tractable and intractable cases of MTO while sticking to optimal (instead of approximate) solutions. In particular, our focus is on the "amount of signal flow" over vertices and edges, respectively, and how this influences the computational complexity of MTO.

• We show that W-MTO can be solved in $O\left({\text{2}}^{m_{v}}\cdot |P|+n^{\text{4}}\right)$ time on an *n*-vertex tree, where *m_v _*denotes the maximum number of connecting paths (one-to-one corresponding to the input vertex pairs) over any tree vertex. In other words, W-MTO is fixed-parameter tractable with respect to the parameter *m_v_*.

• We introduce the concept of cross pairs and show that cross-pair-free instances of W-MTO can be solved in quadratic time, as a corollary also improving the cubic-time algorithm of Medvedovsky et al. [3] for MTO on paths to quadratic time.

• We additionally show that W-MTO is fixed-parameter tractable with respect to the parameter *q_v _*which is the maximum number of cross pairs over any vertex; namely, it can be solved in $O\left({\text{2}}^{q_{v}}\cdot n^{2}\cdot q_{v}\right)$ time.

• Shifting the focus from "maximum vertex signal flow" to "maximum edge signal flow", we show a sharp complexity dichotomy: W-MTO can be solved in linear time if no tree edge has to carry more than two signals, but if this maximum edge signal flow is three, MTO already becomes NP-hard.

• Finally, we briefly discuss some practical aspects of exactly solving the so far very few considered real-world instances and conclude that these can be already solved to optimality within milliseconds (via at least three different strategies). However, we also make the point that with the future availability of further real-world data, our new algorithms could be of significant practical relevance beyond so far known or straightforward approaches.

## Preliminaries, Basic Facts, and Simple Observations

For ease of presentation, for a W-MTO instance (*T*, *P*, *ω*), we always assume that *ω*([*s*, *t*]) = 0 for all pairs *s*, *t *∈ *V *with [*s*, *t*] ∉ *P*. Moreover, subsequently mostly referring to MTO, the presented concepts and definitions clearly apply to W-MTO as well. Note that in a tree *T *= (*V*, *E*), for each ordered pair [*a*, *b*] of vertices, there exists a uniquely determined path connecting these vertices. We will therefore often write *the path defined by the pair *[*a*, *b*] when we refer to the unique path in the tree starting in vertex *a *and ending in vertex *b*, or talk about pairs and paths interchangeably. Sometimes, we also talk about paths in the tree which do not necessarily correspond to pairs. We denote the undirected path connecting vertices *v *and *w *in *T *by path_*T *_(*v*, *w*). Moreover, *P_v_*: = {[*s*, *t*] ∈ *P *| *v *∈ *V *(path_*T *_(*s*, *t*))} denotes the set of paths *passing through a vertex v *(note that this includes paths of which *v *is an endpoint). An MTO instance is called *rooted *if the underlying tree *T *is rooted. In a rooted tree *T *= (*V*, *E*), if vertex *a *∈ *V *is an ancestor of vertex *b *∈ *V*, then we use the notation *a *≺ *b*. The subtree of *T *rooted at *v *∈ *V *is denoted *T_v_*.

Let (*T *= (*V*, *E*), *P*) be an MTO instance, and let *x*, *y *∈ *P *be two pairs. We say that *x conflicts with y *if there exists no orientation of *T *for which both *x *and *y *are satisfied. From an *n*-vertex MTO instance, we build a so called *conflict graph *in which each vertex corresponds to an input pair of the MTO instance, and where there is an edge between two pairs if and only if they conflict with each other. More formally, given an MTO instance (*T *= (*V*, *E*), *P*), the corresponding conflict graph *G_c_*(*T*, *P*) is defined *G_c_*(*T*, *P*):= (*P*, *E_c_*) where *E_c _*: = {{*u*, *v*} | *u*, *v *∈ *P *∧  *u *conflicts with *v*}.

The computation of the conflict graph can be done in Θ(*n*^4^) time. It clearly cannot be done faster, because up to *O*(*n*^4^) conflicts are possible. To achieve the desired bound, we thus need to decide in constant time whether two pairs conflict with each other. This is done using an appropriate data structure and two simple observations: First, in a rooted tree, least common ancestors (LCAs) can be calculated in constant time after some linear time preprocessing [12]. Second, two pairs are in conflict if and only if their paths run in different directions through an edge incident on the lower one of the two LCAs of the two pairs. Clearly, for an orientation of (*T*, *P*), in *G_c _*there are no edges (that is, conflicts) between the vertices corresponding to the satisfied source–target pairs, and hence the vertices corresponding to the non-satisfied source–target pairs form a vertex cover for *G_c_*, that is, a vertex set *V' *⊆ *P *such that for every edge *e *∈ *E_c _*at least one endpoint of *e *is in *V'*. This yields the following useful observation.

**Proposition 1. ***Finding a minimum-weight vertex cover in the conflict graph G_c_*(*T*, *P*) *one-to-one corresponds to determining a minimum-weight set of pairs that cannot be satisfied in *(*T*, *P*).

It is generally assumed that the fact that a problem is NP-hard implies that there is no algorithm that finds an optimal solution and has running time bounded by a polynomial of the size of the input. Parameterized complexity is a *two-dimensional *framework for the analysis of computational complexity [13-15]. One dimension is the input size *n*, and the other one is the *parameter *(usually a positive integer). A problem is called *fixed-parameter tractable *(fpt) with respect to a parameter *x *if it can be solved in *f*(*x*) *⋅ n^O^*^(1)^ time, where *f *is a computable function only depending on *x*. If a problem is fixed-parameter tractable with respect to *x*, we can hope for efficient optimal solutions as long as the parameter is not too large. Due to Proposition 1 we can immediately conclude that MTO and W-MTO are fixed-parameter tractable with respect to the parameter "number of pairs" *p*, since the conflict graph has *p *vertices and we can find a minimum-weight vertex cover by trying all possibilities in 2*^p ^⋅ n^O^*^(1)^ time. Further, since minimum-weight vertex covers can be found in *O*(1*.*379*^k^*+ *kn*) time [16], we have fixed-parameter tractability with respect to the parameter "number of unsatisfied pairs", and if all weights are at least one, also with respect to the parameter "total weight of unsatisfied pairs".

Tree-decomposition-based algorithms have been successfully applied in the area of computational biology, for instance, in the context of structure–sequence alignment [17]. Informally speaking, the *treewidth *[15] measures the "tree-likeness" of a graph, and a tree decomposition is the "embedding" of a graph into a tree depicting the tree-like structure of the graph.

We recall the following definitions from literature [18]: A *tree decomposition *of a graph *G *= (*V*, *E*) is a pair $\langle \left\{X_{i}|i\in I\right\},T\rangle$, where each *X_i _*is a subset of *V *called *bag*, and T=(I,F) is a tree with node set *I *and edge set *F*. The following must hold:

1. ${⋃}_{i\in I}X_{i}=V$;

2. for every edge {*u*, *v*} ∈ *E*, there is an *i *∈ *I *such that {*u*, *v*} ⊆ *X_i_*; and

3. for all *i*, *j*, *l *∈ *I*, if *j *lies on the path between *i *and *l *in *T*, then *X_i _*∩ *X_l _*⊆ *X_j_*.

The *width *of $\langle \left\{X_{i}|i\in I\right\},T\rangle$, is max{|*X_i_*| | *i *∈ *I*} - 1. The *treewidth *of *G *is the minimum width over all tree decompositions of *G*.

## Methods and Results

### Bounded Signal Flow Over Vertices

In this subsection, we investigate how the vertex-wise structure of the source–target pairs influences the computational complexity of MAXIMUM TREE ORIENTATION. More specifically, first we consider the parameter *m_v _*denoting the maximum number of source–target paths passing through a vertex. We show that MTO can be solved in *O*(2*^mv^*⋅ |*P*| + *n*^4^) time. In other words, MTO is fixed-parameter tractable with respect to the parameter *m_v_*. Motivated by this positive result, we explore in more depth the structure of the source–target paths that pass through a vertex. To this end, we introduce the concept of "cross pairs" and show that for cross-pair-free instances MTO can be solved in *O*(*n*^2^) time. Informally speaking, an instance is cross-pair-free if the input tree can be rooted such that for each source–target pair one endpoint is an ancestor of the other one. Then, for a rooted MTO instance a cross pair is a source–target pair such that none of its endpoints is the ancestor of the other endpoint. By refining the solving strategy for cross-pair-free instances, we show that MAXIMUM TREE ORIENTATION can be solved in $O\left({\text{2}}^{q_{v}}\cdot n^{2}\cdot q_{v}\right)$ time, where *q_v _*denotes the maximum number of cross pairs passing through a vertex.

All algorithms in this subsection are based on dynamic programming, and, hence, since source–target pair weights can easily be incorporated, extend to W-MTO.

#### Parameter "Maximum Number of Pairs Per Vertex"

Here, we show that W-MTO is fixed-parameter tractable for the parameter *m_v _*denoting the maximum number of source–target pairs passing through a vertex. To this end, we prove in Theorem 1 that we can construct in polynomial time a tree decomposition of the conflict graph of treewidth at most *m_v_*. Recall that (weighted) MTO is equivalent to (weighted) VERTEX COVER on the conflict graph (see Proposition 1). Thus, the running time follows by the fact that (weighted) VERTEX COVER can be solved in *O*(2^tw^*n*) time, given a tree decomposition of width tw [15].

**Theorem 1. ***On n-vertex trees*, WEIGHTED MAXIMUM TREE ORIENTATION*is solvable in O*(2*^m^v *⋅ |*P*| + *n*^4^) *time, where m_v _denotes the maximum number of source–target pairs passing through a vertex*.

*Proof*. First, we show how to construct a tree decomposition of width *m_v _*of the conflict graph in polynomial time. Let (*T *= (*V*, *E*), *P*) denote an MTO instance and let *G_c _*= (*P*, *E_c_*) denote the associated conflict graph. The basic idea is that we can use *T *as the underlying tree of a tree decomposition of *G_c _*= (*P*, *E_c_*). More specifically, the tree decomposition is given by 〈{*P_v _*| *v *∈ *V*}, *T*〉 for all *v *∈ *V*. Recall that *P_v _*denotes the set of source–target pairs passing through *v*. Observe that each vertex *p *∈ *P *of the conflict graph appears exactly in the bags *X_v _*for all *v *∈ *V *(path_*T*_(*p*)). Moreover, note that if two source–target pairs *p *= [*s*, *t*] and *p*' = [*s*', *t*'] are in conflict (and hence are adjacent in the conflict graph), then path_*T *_(*s*, *t*) and path_*T *_(*s*', *t*') have at least one edge and thus at least two vertices in common. Hence, every edge of the conflict graph is contained in at least one of the *X_v_*'s. Thus, all conditions of a tree decomposition are fulfilled. Moreover, the width of this tree decomposition is clearly *m_v _*- 1. The conflict graph, the sets *P_v_*, and the tree decomposition can be computed in *O*(*n*^4^) time. Thus, the overall running time follows by the fact that WEIGHTED VERTEX COVER can be solved in *O*(2^tw ^|*P*|) time, given a tree decomposition of width tw of *G_c _*[15].

#### Cross Pairs

In Theorem 1, we have shown that W-MTO is fixed-parameter tractable with respect to the parameter *m_v_*. In the following, will strengthen this result by showing that W-MTO is fixed-parameter tractable with respect to the parameter "number of a special type of source–target pairs (the so-called cross pairs) passing through a vertex". The idea is to identify a "trivial" (that is, polynomial-time solvable) special case of the problem and then to investigate instances that are close to these trivial instances, their closeness measured in terms of a certain parameter which is referred to as *distance from triviality *[19,20].

In the following, we will always consider *rooted *trees. Informally speaking, a cross-pair-free instance only contains source–target pairs whose corresponding paths are directed either towards the root or towards the leaves, but do not change their direction. Cross-pair-free instances of W-MTO are of special interest since they constitute our "trivial instances".

**Definition 1**. *Let *(*T *= (*V*, *E*), *P*, *ω*) *be an instance of W-MTO where T is a rooted tree. A source–target pair p *= [*a*, *b*] ∈ *P is called cross pair if neither a is an ancestor of b nor b an ancestor of a. An instance of W-MTO is called cross-pair-free if T can be rooted such that P does not contain any cross pairs*.

#### Cross-pair-free Instances

Now, we devise a dynamic-programming-based algorithm solving W-MTO in quadratic time on cross-pair-free instances.

**Theorem 2**. *On n-vertex trees*, WEIGHTED MAXIMUM TREE ORIENTATION*for cross-pair-free instances with given root can be solved in O*(*n*^2^) *time*.

*Proof*. Algorithm. We present a dynamic programming algorithm with quadratic running time solving a cross-pair-free W-MTO instance (*T *= (*V*, *E*), *P*, *ω*) with root *r*. For the presentation of the algorithm, we use the following notation. For *v *∈ *V*, let *T_v _*be the subtree of *T *rooted at *v*. For all *v*, *w *∈ *V *with *v *≺ *w *(that is, *v *is an ancestor of *w*) let $T_{w}^{v}$ denote the subtree of *T *induced by $V_{w}^{v}:=V\left(T_{w}\right)\cup V\left(\text{pat}{\text{h}}_{T}\left(v,w\right)\right)$.

For ease of presentation, let $V_{w}^{w}:=V\left(T_{w}\right)$. Moreover, let $P_{w}^{v}:=\left\{\left[s,t\right]\in P|s,t\in V_{w}^{v}\right\}$. That is, $T_{w}^{v}$ is the tree consisting of the path path_*T *_(*v*, *w*) and the subtree *T_w _*rooted at *w*, and $P_{w}^{v}$ are the pairs with both endpoints in $T_{w}^{v}$. Finally, the *weight *of an orientation ${\vec{T}}_{w}^{v}$ of $\left(T_{w}^{v},P_{w}^{v}\right)$ is the sum of the weights of the pairs in $P_{w}^{v}$ satisfied by ${\vec{T}}_{w}^{v}$.

The algorithm maintains an *n *× *n *dynamic programming table *S*, containing for each *v*, *w *∈ *V *with *v *≺ *w *or *v *= *w *the two entries *S*(*v*, *w*) and *S*(*w*, *v*). The goal of the dynamic programming procedure is to fill *S *in accordance with the following definition.

For all *v*, *w *∈ *V *with *v *≺ *w*, entry *S*(*v*, *w*) is the maximum weight of an orientation of $\left(T_{w}^{v},P_{w}^{v}\right)$ among all orientations of $\left(T_{w}^{v},P_{w}^{v}\right)$ orienting the path between *v *and *w *from *v *to *w *(that is, away from the root). Analogously, *S*(*w*, *v*) is the maximum weight of an orientation of $\left(T_{w}^{v},P_{w}^{v}\right)$ among all orientations of $\left(T_{w}^{v},P_{w}^{v}\right)$ orienting the path between *v *and *w *from *w *to *v *(that is, towards the root). Note that in the case *v *= *w *we have that *S*(*v*, *v*) is the weight of an optimal orientation of the subtree rooted at *v*.

Next, we describe how our algorithm computes the entries of *S *in accordance with this definition. The weight of an optimal orientation of (*T*, *P*) can then be found in *S*(*r*, *r*).

To compute the entries of *S*, visit all vertices *w *∈ *V *in a bottom-up traversal. Then, for each *w *consider all vertices *v *∈ *V *with *v *= *w *or *v *≺ *w *and set (omit the sum if *w *is a leaf):

(1) $$\begin{array}{c} S\left(v,w\right):=A\left(v,w\right)\\ \;\;\;\;\;\;+\underset{u\;\text{is}\;\text{a}\;\text{child}\;\text{of}\;w}{\sum }\text{max}\left\{S\left(u,w\right),S\left(v,u\right)-A\left(v,w\right)\right\}, \end{array}$$

(2) $$\begin{array}{c} S\left(w,v\right):=A\left(w,v\right)\\ \;\;\;\;\;\;\;+\underset{u\;\text{is}\;\text{a}\;\text{child}\;\text{of}\;w}{\sum }\text{max}\left\{S\left(w,u\right),S\left(u,v\right)-A\left(w,v\right)\right\}. \end{array}$$

Herein, *A *(*v*, *w*) denotes the sum of the weights of the source–target pairs with both endpoints on path_*T *_(*v*, *w*) that are satisfied when orienting the path between *v *and *w *from *v *to *w*, that is,

(3) A(v,w):=ω({[s,t]∈P|   s,t∈V(pathT(v,w))^s≺t}).

Analogously, *A *(*v*, *w*):= *ω *({[*s*, *t *∈ *P *| *s*, *t *∈ *V *(path_*T *_(*v*, *w*)) ^ *t *≺ *s*}). Moreover, for ease of presentation we assume that *A *(*v*, *v*) = 0.

## Correctness

For the correctness of the algorithm note the following. For a leaf *w *and an ancestor *v *of *w*, the tree $T_{w}^{v}$ is identical to the path path_*T *_(*v*, *w*). Hence, the sum of the weights of pairs that can be satisfied by orienting the path either from *v *to *w *or from *w *to *v *is *A*(*v*, *w*) and *A*(*w*, *v*), respectively. Next, consider the case that *w *is an inner vertex and let *v *be an ancestor of *w*. Moreover, let *u*_1_,..., *u_l _*denote the children of *w*. We argue that the maximum weight of an orientation of $\left(T_{w}^{v},P_{w}^{v}\right)$ orienting the edges on path_*T *_(*v*, *w*) towards *w *equals

(4) $$A\left(v,w\right)+\overset{\ell }{\underset{i=1}{\sum }}\text{max}\left\{S\left(u_{i},w\right),S\left(v,u_{i}\right)-A\left(v,w\right)\right\},$$

and, hence, *S*(*v*, *w*) is computed correctly. To this end, consider a maximum-weight orientation ${\vec{T}}_{w}^{v}$ of $\left(T_{w}^{v},P_{w}^{v}\right)$ orienting the edges on path_*T *_(*v*, *w*) towards *w*. If, for a child *u_i_*, ${\vec{T}}_{w}^{v}$ contains the arc (*u_i_*, *w*), then the contribution of the source–target pairs in $P_{w}^{v}$ with at least one endpoint in $T_{u_{i}}$ to the weight of ${\vec{T}}_{w}^{v}$ is *S*(*u_i_*, *w*); note that no source–target pair of $P_{w}^{v}$ with exactly one endpoint in $T_{u_{i}}^{w}$ is satisfied by ${\vec{T}}_{w}^{v}$, and thus the contribution of these pairs is *S *(*u_i_*, *w*) (a smaller contribution would contradict the optimality of ${\vec{T}}_{w}^{v}$). Moreover, if for a child *u_i _*the oriented tree ${\vec{T}}_{w}^{v}$ contains the arc (*w*, *u_i_*), then it follows by a similar argument that the contribution of the paths in $P_{w}^{v}$ with at least one endpoint in $V\left(T_{u_{i}}\right)$ is *S*(*v*, *u_i_*) - *A*(*v*, *w*). The only difference is that the contribution of the source–target pairs with both endpoints in *V *(path_*T *_(*v*, *w*)) is already considered in the above formula, and, hence, must be subtracted from *S*(*v*, *u_i_*).

**Running time**. For the running time bound, we show that A can be computed in *O*(*n*^2^) time in a preprocessing step. Then, the overall running time is clearly bounded by

(5) $$O\left(\sum_{v\in V}\sum_{w\in V}{\text{deg}}_{T}(w)\right)=O\left(\sum_{v\in V}n\right)=O(n^{2}),$$

since ∑*_w∈V _*deg_*T *_(*w*) = 2(*n *- 1) in trees. Clearly for *v*, *w *∈ *V *with *v *≺ *w *the matrix entries *A*(*v*, *w*) and *A*(*w*, *v*) can be computed by setting

(6) A(v,w):=ω([v,w])+A(v,y)+A(x,w)-A(x,y)

and

(7) A(w,v):=ω([w,v])+A(w,x)+A(y,v)-A(y,x),

where *x *is the neighbor of *v *and *y *is the neighbor of *w *on path_*T *_(*v*, *w*). This assumes, however, that all the entries of *A *for pairs with distance *l *- 1 are known before computing the entries of the pairs with distance *l*. This can be ensured by using a queue (first-in-first-out data structure) as follows. For the computation of *A*, first, for all edges {*v*, *w*} ∈ *E *with *v *≺ *w *set *A*(*v*, *w*):= *ω*([*v*, *w*]) and *A*(*w*, *v*):= *ω*([*w*, *v*]) (let *ω*([*s*, *t*]):= 0 if [*s*, *t*] ∉ *P*) and append the pair (*v*, *w*) at the tail of the queue. After each edge has been processed, proceed as follows. Until the queue is empty, let (*x*, *w*) denote the next element at the head of the queue and let *v *denote the parent of *x *and let *y *denote the parent of *w *in *T *(if *x *= *r *remove (*x*, *w*) from the head position of the queue and do nothing else). It is easy to verify that the entries for the pairs (*x*, *w*) and (*v*, *y*) have already been computed and, hence, can be used to compute *A*(*v*, *w*) and A(*w*, *v*) as described above. Finally, we remove (*x*, *w*) from the head position of the queue and append (*v*, *w*) at the tail of the queue. Clearly, for every pair *v*, *w *of vertices with *v *≺ *w*, we need a constant number of operations to compute the two table entries *A*(*v*, *w*) and *A*(*w*, *v*), resulting in an overall running time bound of *O*(*n*^2^).

Note that if the root of a cross-pair-free W-MTO instance is not known, it can be calculated in *O*(*n*|*P*|) time by trying all roots and then checking for each pair if the least common ancestor is one of the two endpoints.

As an immediate consequence of Theorem 2, we can improve the cubic-time algorithm for MTO on paths by Medvedovsky et al. [3] to quadratic time. Herein, we use that every path rooted at one of its endpoints results in a cross-pair-free instance of MTO.

## Corollary 1

WEIGHTED MAXIMUM TREE ORIENTATION*on n-vertex paths can be solved in O*(*n*^2^) *time*.

## Parameter "Maximum Number of Cross Pairs Passing Through a Vertex"

Next, we show that W-MTO is fixed-parameter tractable with respect to the parameter *q_v _*by extending the dynamic programming algorithm for cross-pair-free instances. Formally, *q_v _*is defined as follows. For a rooted W-MTO instance (*T *= (*V*, *E*), *P*) with root *r*, let *Q *denote the set of cross pairs. Moreover, for *v *∈ *V *let *Q_v _*:= *P_v _*∩ *Q *be the set of cross pairs passing through *v*. With respect to the root *r *the maximum number *q_v_*(*r*) of cross pairs passing through a vertex is given by max_*v∈V *_|*Q_v_*|. Then, *q_v _*is the minimum value of *q_v_*(*r*) over all possible choices *r *to root *T*.

**Theorem 3**.* On n-vertex trees*, WEIGHTED MAXIMUM TREE ORIENTATION*with given root can be solved in O(*2*^qv ^⋅ q_v _⋅ n*^2^*) time, where q_v _denotes the maximum number of cross pairs passing through a vertex*.

*Proof*. The basic idea of the algorithm is to incorporate the cross pairs by trying for every vertex all possibilities to satisfy the cross pairs passing through this vertex. To this end, we extend the matrix *S *by an additional dimension. As a consequence, the dynamic programming update step becomes significantly more intricate.

Let (*T *= (*V*, *E*), *P*, *ω*) be a rooted W-MTO instance with root *r*. For the presentation of the algorithm we use the same notation as in the proof of Theorem 2. In addition, we employ the following definitions. Let *w *∈ *V*. A possibility to satisfy the cross pairs in *Q_w _*is represented by a coloring *c_w _*: *Q_w _*→ {0, 1}, meaning that a cross pair *q *∈ *Q_w _*must be satisfied iff *c_w_*(*q*) = 1. Let *C_w _*denote the set of all 0/1-colorings of *Q_w_*. Note that $|C_{w}|=2^{\left|Q_{w}\right|}$. To incorporate the cross pairs, for every *v*, *w *∈ *V *with *v *≺ *w *or *v *= *w *and for every coloring *c_w_*, the dynamic programming table *S *contains two entries *S*(*v*, *w*, *c_w_*) and *S*(*w, v*, *c_w_*). Informally speaking, *S*(*v*, *w*, *c_w_*) denotes the maximum weight of an orientation of $T_{w}^{v}$ under the assumption that all cross pairs *q *∈ *Q_v _*with *c_w_*(*q*) = 1 are "satisfiable" and the edges in path_*T *_(*v*, *w*) are oriented from *v *towards *w*. Entry *S*(*w*, *v*, *c_w_*) is defined analogously, but here we assume that the edges in path_*T *_(*v*, *w*) are oriented from *w *towards *v*. For a precise description, we use the following notation. Clearly, we are interested only in colorings *c_w _*of *Q_w _*such that any two cross pairs *q*, *q*' ∈ *Q_w _*with *c_w_*(*q*) = *c_w_*(*q*') = 1 are not in conflict. We call such a coloring *locally feasible*. Moreover, we extend the notion "feasible" as follows. As informally described above, we distinguish the two cases that the edges on path_*T *_(*v*, *w*) are oriented

(1) from *v *to *w *(for entry *S*(*v*, *w*, *c_w_*)), or

(2) from *w *to *v *(for entry *S*(*w, v*, *c_w_*)).

For the case (1), a locally feasible coloring *c_w _*is called *feasible *if for each cross pair [*s*, *t*] ∈ *Q_w _*with *c_w_*([*s*, *t*]) = 1 orienting the edges on path_*T *_(*v*, *w*) from *v *to *w *and the edges on path_*T *_(*s*, *t*) from *s *to *t *is simultaneously possible. Analogously, a locally feasible coloring *c_w _*is feasible for case (2) when orienting the edges on path_*T *_(*s*, *t*) from *s *to *t *does not contradict the orientation of the edges on path_*T *_(*v*, *w*) from *w *to *v*.

For a coloring *c_w _*of *Q_w_*, we must ensure that in the considered orientations of $\left(T_{w}^{v},P_{w}^{v}\right)$ all cross pairs *q *∈ *Q_w _*with *c_w_*(*q*) = 1 are satisfiable. Therefore, we call an orientation of $\left(T_{w}^{v},P_{w}^{v}\right)$* consistent with a cross pair *[*s*, *t*] ∈ *Q_w _*(note that $\left[s,t\right]\in P\backslash P_{w}^{v}$ is allowed) if the common edges of $T_{w}^{v}$ and path_*T *_(*s*, *t*) are oriented from *s *to *t*. Finally, we call an orientation of $\left(T_{w}^{v},P_{w}^{v}\right)$* consistent with a coloring c_w _*of *Q_w _*if the orientation is consistent with each cross pair *q *∈ *Q_w _*with *c_w_*(*q*) = 1.

With these notations, we can formally define the meaning of the entries of *S*. For every two vertices *v*, *w *∈ *V *with *v *≺ *w *or *v *= *w *and for every 0/1-coloring *c_w _*∈ *Q_w _*the entry *S*(*v*, *w*, *c_w_*) is -∞ if the coloring *c_w _*is not feasible for case (1). Otherwise, *S*(*v*, *w*, *c_w_*) denotes the maximum weight of an orientation of $\left(T_{w}^{v},P_{w}^{v}\right)$ among all orientations of $\left(T_{w}^{v},P_{w}^{v}\right)$ fulfilling the following constraints:

• the edges on path_*T *_(*v*, *w*) are oriented from *v *to *w*, and

• the orientation is consistent with *c_w_*.

This definition ensures that the orientation is not conflicting with the realization implied by the coloring *c_w _*and the fixed orientation of path_*T *_(*v*, *w*). The entry *S*(*w, v*, *c_w_*) is defined analogously with the difference that here we assume that the edges on path_*T *_(*v*, *w*) are oriented from *w *to *v*. Note that the cross pairs having only one endpoint in $T_{w}^{v}$ are not contained in $P_{w}^{v}$, and hence do not contribute to the weight of an orientation of $\left(T_{w}^{v},P_{w}^{v}\right)$. Observe that ${max}_{c_{r}\in C_{r}}S(r,r,c_{r})$ is the maximum weight of an orientation of the whole instance (*T*, *P*, *ω*) since $T_{r}^{r}=T$ and we build the maximum over all colorings of the cross pairs in *Q_r_*. Next, we provide a strategy to compute the entries of *S *in accordance with this definition. In the update step, we need to adjust the tables of a vertex *w *with the tables of its children. Doing so, we have to ensure that we only consider colorings that are not contradictory to each other. Let *c_u _*denote a coloring of *Q_u _*and *c_w _*denote a coloring of *Q_w_*. We use *c_u_*|*c_w _*to denote that *c_u _*and *c_w _*agree in the coloring of the cross pairs in *Q_u _*∩ *Q_w_*, that is, for all *q *∈ *Q_u _*∩ *Q_w _*it holds that *c_u_*(*q*) = *c_w_*(*q*). Finally, let W_LCA_(*w*, *c_w_*) denote the sum of the weights of the cross pairs [*s*, *t*] ∈ *Q_w _*with *c_w_*([*s*, *t*]) = 1 that have *w *as their least common ancestor.

For the computation of *S*, visit each vertex *w *∈ *V *in a post-order traversal of *T*. For each *w *consider all vertices *v *∈ *V *with *v *≺ *w *or *v *= *w*. Moreover, let *u*_1_, ..., *u_l _*denote the children of *w *(if *w *is not a leaf). Then, for each coloring *c_w _*∈ *C_w_*, set *S*(*v*, *w*, *c_w_*):= -∞ if *c_w _*is infeasible for case (1), otherwise set (omit the sum if *w *is a leaf)

(8) $$\begin{array}{c} S\left(v,w,c_{w}\right):=A\left(v,w\right)+W_{\text{LCA}}\left(w,c_{w}\right)\\ \;\;\;\;\;\;\;\;+\overset{\ell }{\underset{i=1}{\sum }}M\left(u_{i},v,w,c_{w}\right) \end{array}$$

where

(9) $$\begin{array}{l} M(u_{i},v,w,c_{w}):=max\{\\ max\{S(v,u_{i},c_{u_{i}})-A(v,w):c_{u_{i}}\in C_{u_{i}},c_{u_{i}}\;|\;c_{w}\},\\ max\{S(u_{i},w,c_{u_{i}}):c_{u_{i}}\in C_{u_{i}},c_{u_{i}}\;|\;c_{w}\}\}. \end{array}$$

and *S*(*w*, *v*, *c_w_*):= -∞ if *c_w _*is infeasible for case (2), otherwise set (omit the sum if *w *is a leaf)

(10) $$\begin{array}{c} S\left(w,v,c_{w}\right):=A\left(w,v\right)+W_{\text{LCA}}\left(w,c_{w}\right)\\ \;\;\;\;\;\;\;\;+\overset{\ell }{\underset{i=1}{\sum }}M\left(u_{i},w,v,c_{w}\right) \end{array}$$

where

(11) $$\begin{array}{l} M(u_{i},w,v,c_{w}):=max\{\\ max\{S(u_{i},v,c_{u_{i}})-A(w,v):c_{u_{i}}\in C_{u_{i}},c_{u_{i}}\;|\;c_{w}\},\\ max\{S(w,u_{i},c_{u_{i}}):c_{u_{i}}\in C_{u_{i}},c_{u_{i}}\;|\;c_{w}\}\}. \end{array}$$

Herein, *A *is defined exactly as in the proof of Theorem 2.

**Correctness**. For the correctness, we argue that for *v*, *w *∈ *V *with *v *≺ *w *and for a coloring *c_w _***∈ ***C_w _*that is feasible for case (1), the maximum weight of an orientation of $\left(T_{w}^{v},P_{w}^{v}\right)$ consistent with *c_w _*that orients the edges on path_*T *_(*v*, *w*) from *v *to *w *is $A\left(v,w\right)+W_{\text{LCA}}\left(w,c_{w}\right)+\sum_{i=1}^{\ell }M\left(u_{i},v,w,c_{w}\right)$, and, hence, *S*(*v*, *w*, *c_w_*) is computed correctly. To this end, first consider the case that *w *is a leaf. Then, $T_{w}^{v}$ is identical to path_*T *_(*v*, *w*) and *Q_w _*= ∅. Hence, in this case exactly the source–target pairs $\left[s,t\right]\in P\backslash P_{w}^{v}$ with *s *≺ *t *are satisfied whose total weight per definition is *A*(*v*, *w*). Second, consider the case that *w *is an internal vertex with children *u*_1_, ..., u_l_. Moreover, let ${\vec{T}}_{w}^{v}$ be an optimal orientation consistent with *c_w _*that orients the edges on path_*T *_(*v*, *w*) from *v *to *w*. Assume that this orientation contains for a child *u_i _*the arc (*u_i_*, *w*). Then, with respect to *w *and *u_i_*, the subgraph of ${\vec{T}}_{w}^{v}$ induced by the vertices in $V_{u_{i}}^{w}$ is an orientation of $T_{u_{i}}^{w}$ consistent with a coloring $c_{u_{i}}$ that clearly agrees with *c_w_*. Thus, the contribution of *u_i _*to the weight of ${\vec{T}}_{w}^{v}$ is the maximum $S\left(u_{i},w,c_{u_{i}}\right)$ over all $c_{u_{i}}\in Q_{u_{i}}$ that agree with *c_w_*. Similarly, if ${\vec{T}}_{w}^{v}$ contains the arc (*w*, *u_i_*) for a child *u_i_*, the subgraph of ${\vec{T}}_{w}^{v}$ induced by the vertices in $V_{u_{i}}^{w}$ is an orientation of $T_{u_{i}}^{w}$ consistent with a coloring $c_{u_{i}}$ that clearly agrees with *c_w_*. Thus, the contribution of the pairs in $P_{w}^{v}$ with at least one endpoint in $T_{u_{i}}$ is the maximum of $S\left(v,u_{i},c_{u_{i}}\right)-A\left(v,w\right)$ over all $c_{u_{i}}\in C_{u_{i}}$ that agree with *c_w_* (here, we have to subtract the number of satisfied pairs with both endpoints on path_*T *_(*v*, *w*) that are already accounted for by the term *A*(*v*, *w*) in (8)). Finally, observe that the contribution of the cross pairs *q *in $P_{w}^{v}$ with *c_w_*(*q*) = 1 for which *w *is the least common ancestor are not taken into account in the contributions of the *u_i_*'s. This is done by the term W_LCA _in (8). The argumentation for the correctness of the computation of *S*(*w, v*, *c_w_*) follows analogously.

** Running time**. Next, we analyze the running time. We use the following notation and implementation details. For *w *∈ *V *let $Q_{w}=\left\{p_{1}^{w},\ldots ,p_{n_{w}}^{w}\right\}$. A coloring *c_w _*: *Q_w _*→ {0, 1} is realized by a tuple $\left(c_{1},\ldots ,c_{n_{w}}\right)\in {\left\{0,1\right\}}^{n_{w}}$ with $c_{w}\left(p_{i}^{w}\right)=c_{i}$ for all 1 ≤ *i *≤ *n_w_*. Moreover, the dynamic programming table *S *is realized by two tables $S_{v,w}^{up}$ and $S_{v,w}^{down}$ for every pair *v*, *w *∈ *V *with *v *≺ *w *or *v *= *w *with an entry for every coloring $c\in {\left\{0,1\right\}}^{n_{w}}$ where $S_{v,w}^{up}\left(c\right)=S\left(w,v,c\right)$ and $S_{v,w}^{down}\left(c\right)=S\left(v,w,c\right)$. The table *A *is computed exactly as in the proof of Theorem 2 in *O*(*n*^2^) time in a preprocessing step. Moreover, note that after *O*(*n*) preprocessing time, least common ancestors of the source–target pairs can be found in constant time [12].

To prove the running time, we show that for every pair *v*, *w *with *v *≺ *w *or *v *= *w *the computation in (8) and (10) can be done in $O{(2}^{q_{v}}\cdot q_{v}\cdot {deg}_{T}(w))$ time. We focus on the computation of (8). The running time analysis for (10) follows by the same arguments. The crucial observation is that the summands in the sum in (8) are independent of each other in the sense that the determination of the maximum for one child *u_i _*(the computation of *M*(*u_i_*, *v*, *w*, *c_w_*)) does not depend on the decision made for a different child. Hence, for the computation of the entries of $S_{v,w}^{down}$ proceed as follows. Consider each child *u *of *w *one after another. Let $Q_{w}=\left\{q_{1},\ldots ,q_{s},q_{1}^{\prime },\ldots ,q_{x}^{\prime }\right\}$ and $Q_{u}=\left\{q_{1},\ldots ,q_{s},q_{1}^{\prime \prime },\ldots ,q_{y}^{\prime \prime }\right\}$, that is, {q_1_, ..., *q_s_*} = *Q_w _*∩ *Q_u_*. The crucial point is that we assume that the tables $S_{w,u}^{up}$ and $S_{v,u}^{down}$ are sorted in lexicographical order of the colorings ${\left\{0,1\right\}}^{n_{u}}$. This ensures that the colorings of *u *that agree with a coloring $c\in {\left\{0,1\right\}}^{n_{w}}$ are ordered consecutively in $S_{w,u}^{up}$ and $S_{v,u}^{down}$. Since the tables $S_{w,u}^{up}$ and $S_{v,u}^{down}$ contain each at most $2^{q_{v}}$ entries, the sorting can be achieved in time $O\left(2^{q_{v}}\right)$ using bucket sort. Then, for each fixed *v*, *w*, and *u *all the values *M*(*v*, *w*, *u*, *c*) can be computed in $O\left(2^{q_{v}}q_{v}\right)$ time in one iteration over $S_{w,u}^{up}$ and $S_{v,u}^{down}$ and, hence, for all children of *w *the running time is bounded by $O{(2}^{q_{v}}\cdot q_{v}\cdot {deg}_{T}(w))$. Thus, the overall running time is bounded by

(12) $$O\left(\sum_{v,w\in V}2^{q_{v}}\cdot q_{v}\cdot {deg}_{T}(w)\right)=O{(2}^{q_{v}}q_{v}n^{2}),$$

since *O*(∑*_w∈V _*deg_*T *_(*w*)) = *O*(*n*) in trees.

## Bounded Signal Flow Over Edges

Let *m_e _*be the maximum number of paths that pass through an edge. We consider MTO instances where *m_e _*is limited. We show that the problem is linear-time solvable for *m_e _*≤ 2, but NP-hard for *m_e _*≥ 3, thereby establishing a dichotomy on the complexity of MTO with respect to *m_e_*.

For the polynomial-time algorithm, we employ the following lemma.

**Lemma 1**. *If m_e _*≤ 2, *then the treewidth of G_c_*(*T*, *P*) *is at most two*.

*Proof*. We make use of the following characterization of graphs of treewidth at most two [21]. A graph has treewidth at most two if it can be reduced to the empty graph by the exhaustive application of the following data reduction rules:

(1) deleting vertices of degree 0 or 1,

(2) deleting a degree-2 vertex whose two neighbors are adjacent, and

(3) adding an edge between the two neighbors of a degree-2 vertex *v *if the neighbors are non-adjacent, and subsequently deleting *v*.

We show that if *m_e _*≤ 2, then in the conflict graph *G_c_*(*T*, *P*) we can find a vertex to which one of the above rules applies. Further, we show that the modified smaller conflict graph is still a conflict graph of some MTO instance. Thus, the claim follows by induction.

Clearly, if Rule (1) or (2) applies to a vertex *v *in the conflict graph, then we can just delete the corresponding pair in the MTO instance, and the conflict graph of the resulting MTO instance is identical to the graph that results by deleting *v*.

Next, we show that if neither Rule (1) nor Rule (2) applies, then we can find a vertex *v *in the conflict graph to which Rule (3) applies. To this end, let (*T*, *P*) with *T *= (*V*, *E*) denote an MTO instance and assume that *T *is rooted at an arbitrarily chosen inner vertex *r*. Moreover, among all vertices that are the least common ancestors of a pair in *P*, let *x *be one with maximum distance to the root *r *(that is, a deepest least common ancestor). We distinguish two cases based on whether *x *is an endpoint of a pair with both endpoints in *T_x_*.

First, consider the case that *x *is the endpoint of a pair [*s*, *t*] ∈ *P *with *s*, *t *∈ *V *(*T_x_*). Let *y *be the child of *x *that is contained in the path between *s *and *t*. Observe that by the choice of *x *there is no pair with both endpoints in *T_y_*. Hence, for every pair that is in conflict with [*s*, *t*], the corresponding path contains the edge {*x*, *y*}. Thus, since *m_e _*= 2, the pair [*s*, *t*] is in conflict with at most one other pair, and therefore the corresponding vertex has degree at most one in the conflict graph: a contradiction to the fact that neither Rule (1) nor (2) apply.

**Figure 1:**
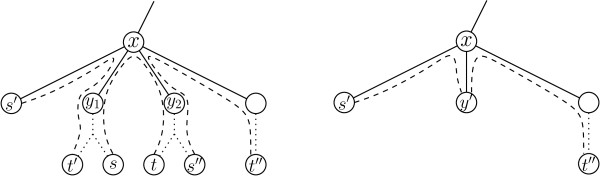
**Applicability of reduction**. Left: Illustration of the structure for a deepest least common ancestor *x *of the pairs as described in the proof of Lemma 1. The dashed lines represent the paths between the endpoints of a pair. If [*s*, *t*] is in conflict with two other pairs, then these pairs must be conflicting with [*s*, *t*] in the edges {*x*, *y*_1_} and {*x*, *y*_2_} since *x *a deepest least common ancestor. Right: Illustration of the replacement of the subtrees rooted at *y*_1 _and *y*_2 _by a single vertex *y*'. Note that (*s*', *y*') and (*y*', *t*'') are in conflict.

Second, consider the case that *x *is not an endpoint of any pair with both endpoints in *V*(*T_x_*). Moreover, let *p *= [*s*, *t*] ∈ *P *be an arbitrarily chosen pair with *s*, *t *∈ *V *(*T_x_*). Let *y*_1 _and *y*_2 _denote the two children of *x *such that (without loss of generality) *s *∈ *V *(*T_y1_*) and *t *∈ *V *(*T_y2_*). Let *v*_[*s*,*t*] _denote the vertex of *G_c _*corresponding to [*s*, *t*]. First, note that by the assumption that Rule (1) does not apply, *v*_[*s*,*t*] _has degree at least two. Moreover, by the choice of *x *there is no pair with both endpoints in *V *(*T_y1_*) or in *V *(*T_y2_*). Thus, every pair that is in conflict with [*s*, *t*] uses either the edge {*x*, *y*_1_} or the edge {*x*, *y*_2_}. Thus, since *m_e _*≤ 2 and $\deg_{G_{c}}\left(v_{\left[s,t\right]}\right)\geq 2$, there are exactly two pairs *p*' = [*s*', *t*'], *p*'' = [*s*'', *t*''] ∈ *P *that are in conflict with [*s*, *t*]. Assume without loss of generality that *t*' ∈ *V *(*T_y1_*) and *s*'' ∈ *V *(*T_y2_*) (see Figure 1 for an illustration). Since Rule (2) does not apply to *v*_[*s*,*t*]_, we can assume that *p*' and *p*'' are not in conflict with each other. Hence, Rule (3) can be applied to *v*_[*s*,*t*]_. Let $G_{c}^{\prime }$ denote the graph that results by first making the two neighbors of *v *adjacent and subsequently deleting *v*. It remains to show how to transform the MTO instance such that the conflict graph of the new instance is identical to $G_{c}^{\prime }$. To this end, consider the MTO instance that results by deleting the vertices in *V *(*T_y1_*) ∪ *V *(*T_y2_*), removing the pairs [*s*, *t*], [*s*', *t*'], and [*s*'', *t*''] and subsequently adding a vertex *y*', making *y*' adjacent to *x*, and adding the pairs [*s*', *y*'] and [*y*', *t*''] (see Figure 1 for an illustration). Clearly, [*s*', *y*'] and [*y*', *t*''] are in conflict. Moreover, since only the pairs *p*, *p*', and *p*'' have endpoints in *V *(*T_y1_*) ∪ *V *(*T_y2_*), this transformation does not change the conflicts with the other pairs. Further, we have that *m_e _*≤ 2 in the resulting MTO instance.

Since width-two tree decompositions can be constructed in linear time [21] and weighted VERTEX COVER can be solved in linear time on graphs with constant treewidth [15], this yields linear-time solvability for WEIGHTED MAXIMUM TREE ORIENTATION with *m_e _*≤ 2.

**Theorem 4**. *If m_e _*≤ 2, *then *WEIGHTED MAXIMUM TREE ORIENTATION*can be solved in linear time*.

*Proof*. To be able to determine the path between a pair [*s*, *t*] in *O*(*n*) time, we root the tree arbitrarily and calculate in linear time a data structure that allows least common ancestor queries in constant time [12]; the path can then be found by going upwards from *s *and *t *until hitting their least common ancestor, and then joining the two partial paths. We then construct the conflict graph by marking for each path the corresponding edges with the pair and the direction, and then registering a possible conflict for each tree edge. Since there can be only linearly many markings and conflicts, the construction takes *O*(*n*) time. A tree decomposition of width two can then be found in linear time [21], and, as mentioned above, solving weighted VERTEX COVER on a graph with treewidth at most two takes only linear time, too [15].

We can further prove that for *m_e _*≥ 3, MTO is NP-hard even on stars, that is, on trees where all leaves are attached to the same vertex. The proof is by reduction from MAXDICUT.

**Theorem 5**. MAXIMUM TREE ORIENTATION*on stars with m_e _*≥ 3 *is NP-complete*.

*Proof*. As Medvedovsky et al. [3] pointed out, the NP-hard MAXDICUT problem, defined as follows, can be reduced to MTO on stars.

MAXDICUT

Given a directed graph G = (*V*, *A*) and a nonnegative integer *k*, is it possible to find a subset of vertices *C *⊆ *V *such that there are at least |*A*| - *k *arcs (*v*, *w*) ∈ *A *with *v *∈ *C *and *w *∉ *C*?

From a MAXDICUT instance (*G *= (*V*, *A*), *k*), one constructs an equivalent MTO instance (*T *= (V', *E*), *P*, *k*) by setting V':= *V *∪ {*r*}, *E *:= {{*v*, *r*} | *v *∈ *V*}, and *P *:= *A*, where *r *∉ *V *is a new root vertex [3]. Clearly, if a MAXDICUT instance has maximum degree three, then it reduces to an MTO instance with *m_e _*≤ 3. Thus, it remains to show that MAXDICUT with maximum degree three is NP-hard. (Unfortunately, there seems to be no apt reduction from the undirected version MAXCUT, which is NP-hard for maximum degree three [22].)

MAXDICUT can also be formulated as the problem to delete up to *k *arcs to obtain a graph where every vertex is only startpoint or only endpoint of arcs. We can characterize such graphs by a forbidden substructure consisting of three vertices *u*, *v*, *w *connected by the arcs (*u*, *v*) and (*v*, *w*) (the arcs (*u*, *w*) and (*w*, *u*) may or may not be present). Thus, if we ignore graphs with multiple arcs between two vertices, we have three forbidden induced subgraphs on three vertices. In this way, MAXDICUT is similar to the TRANSITIVITY DELETION problem [23], which given a directed graph, asks for up to *k *arc deletions to make it *transitive*, that is, to fulfill for all *u*, *v*, *w *∈ *V *that (*u*, *v*) ∈ *A *∧ (*v*, *w*) ∈ *A *⇒ (*u*, *w*) ∈ *A*. Transitive graphs are characterized by two of the three forbidden subgraphs for MAXDICUT; the subgraph with {(*u*, *v*), (*v*, *w*), (*u*, *w*)} ⊆ *A *is not forbidden. However, if we examine the directed graphs that are produced in the reduction from 3-SAT that proves NP-hardness of TRANSITIVITY DELETION [23, Sect. 3.1], we notice that this substructure does not occur, and cannot be created by arc deletions. Thus, solving TRANSITIVITY DELETION and MAXDICUT on these directed graphs is equivalent. Since the constructed instances also have degree at most three, we obtain the NP-hardness of MAXDICUT with maximum degree three. It is easy to see that MTO is contained in NP, so we obtain the claimed theorem.

## Observations on Protein Interaction Networks

The goal in this section is to explore the space of practically meaningful parameterizations, here focusing on biological applications. We first performed experiments based on the same data as used by Medvedovsky et al. [3]. The network is a yeast protein-protein interaction network from the Database of Interacting Proteins (DIP) [24], containing 4,737 vertices and 15,147 edges. The cause-effect pairs were obtained from gene knockout experiments by Yeang et al. [2] and contain 14,502 pairs. After discarding small connected components and contracting cycles, we obtained a tree with 1,278 vertices and 5,569 pairs. (These numbers differ slightly from the ones stated by Medvedovsky et al. [3]. We do not use the additional kinase-substrate data, which is only meaningful to evaluate the orientations obtained, and which requires an arbitrary parameter choice not documented by Medvedovsky et al. [3].)

The resulting tree is, as already observed by Medvedovsky et al. [3], very star-like: there is one vertex of degree 1,151 and 1,048 degree-one vertices attached to it. The remaining 229 vertices have degree 1 to 4. All paths connecting cause-effect pairs pass through the central vertex.

We first note that this MTO instance is actually fairly easy to solve exactly. The Integer Linear Program (ILP) by Medvedovsky et al. [3, Sect. 3.1] and VERTEX COVER on the conflict graph solved by either an ILP or a simple branching strategy with data reduction all solve the instance in less than a second. More precisely, the running times are 0.09 s, 0.02 s, and 0.13 s, respectively, on a 2.67 GHz Intel Xeon W3520 machine, using GLPK 4.44 for the ILPs, and with the branching strategy implemented in Objective Caml. The branching strategy finds a vertex *v *of maximum degree and branches into the two cases of taking *v *into the vertex cover or taking all neighbors of *v *into the vertex cover. Before each branch, degree-1 vertices are eliminated by taking their neighbor into the vertex cover. The search in the second branch is cut short when the accumulated vertex cover is larger than that of the first branch.

Note that all three algorithms do not require the parameter *k *(number of unsatisfied pairs) as input, but will determine the minimum *k *such that there is a solution.

The reason that these strategies work so well is probably due to the low value of the parameter *k*: only 77 cause-effect pairs cannot be satisfied. This limits the size of the branch-and-bound tree that underlies all three methods.

In Table 1, we examine several other parameters. Since there are still *p_t _*= 5,569 pairs left after contracting all cycles in the network, using this parameter for a fixed-parameter algorithm seems infeasible. Unfortunately, since all paths run through a single vertex, the parameter *m_v _*is not any more useful. Only about 5% of the pairs are cross pairs after the data reduction, so *q *is already a more promising parameter. However, with a value of *q *= 417, this parameter seems not very helpful. Even if we eliminate pairs that do not conflict with any other pairs, leaving only *n_c _*= 1,287 pairs, we still find at least 306 cross pairs (parameter *q*'). Again, because all paths run through a single vertex, considering cross pairs per vertex does not help here. In summary, for this particular instance the number of unsatisfiable pairs *k *is clearly the most useful parameter.

**Table 1:** Network parameters

Parameter		Value
*n*	Number of network vertices	4,654
*m*	Number of network edges	15,104
*p*	Number of pairs	14,155
*n_t_*	Vertices in MTO instance	1,278
*p_t_*	Number of pairs in MTO instance	5,569
n*	Number of vertices in star	1,049
*m_v_*	Max. number of pairs per vertex	5,569
*m_e_*	Max. number of pairs per edge	371
*q*	Number of cross pairs	417
*q_v_*	Max. number of cross pairs per vertex	417
*q*'	Number of cross pairs after data reduction	306
$q_{v}^{\prime }$	Max. number of cross pairs per vertex after data reduction	306
*n_c_*	Number of vertices in conflict graph	1,287
*m_c_*	Number of edges in conflict graph	4,626
*k*	Number of unsatisfiable pairs	77

Values for various parameters for the protein interaction network instance from Medvedovsky et al. [3].

To examine the effect of the sparseness of the input instance on the various parameters, we investigated another yeast protein interaction network assembled by Nir Yosef from various sources (see references in [25]). In this network, each edge is annotated with a probability of interaction. Thus, by thresholding, we can obtain graphs of different sparseness. The results are shown in Table 2.

**Table 2:** Thresholded network parameters

threshold	*n*	*m*	*p*	*n_t_*	*p_t_*	n*	*m_v_*	*m_e_*	*q*	*q_v_*	*q*'	$q_{v}^{\prime }$	*n_c_*	*m_c_*	*k*
0.000000	5385	39921	14393	799	2014	750	2014	59	7	7	3	3	115	292	17
0.154420	4530	35041	11522	747	2203	705	2203	298	27	27	20	20	475	1632	40
0.371369	4254	32135	10740	796	2443	749	2443	275	47	47	35	35	528	2424	46
0.573290	3871	27128	9445	777	2225	704	2225	268	32	32	13	13	140	311	32
0.573313	2546	8977	5279	638	2311	477	2310	208	252	252	151	151	561	2394	68
0.830093	2206	7136	4346	643	2206	449	2206	192	304	304	193	193	727	4017	83
0.886308	1407	3646	1607	441	787	260	785	45	106	106	88	88	311	1876	75
0.943001	1135	3069	920	361	464	195	463	32	57	57	42	42	179	801	44
0.954421	1039	2504	843	350	489	175	461	45	85	73	71	61	215	3001	81
0.957338	895	2060	681	304	405	119	375	39	64	54	58	50	240	3092	89
0.965986	874	2018	666	299	477	103	411	165	90	78	85	75	358	12284	110
0.984753	668	1676	312	206	163	95	162	20	7	7	6	6	55	222	15
0.989212	581	1322	188	192	167	69	161	86	24	24	24	24	141	1088	32
0.989233	307	681	71	121	70	32	66	36	21	21	11	11	52	219	7
0.990409	294	666	28	114	27	26	26	21	2	2	2	2	9	8	2

Parameters for the largest connected component of the protein interaction network assembled by Nir Yosef [25] with different thresholds for the edge probability. The uneven gaps in the sizes of the instances are because many edges have identical weights.

We see that, here, the parameter *k *is not always a clear winner. When the network becomes sparser, the components that will be shrunk to a single vertex by the cycle contraction will be smaller, leaving fewer pairs with both endpoints on the same tree vertex, and thereby increasing the number of potential conflicts. Only for very high thresholds, the parameter becomes small again, since then the original instance is already much smaller. Still, all instances can be solved in less than one second by the three algorithms mentioned above, which exploit low values of *k*.

We also see that for denser graphs, the parameter values based on the number of cross pairs are quite low, e.g. $q_{v}^{\prime }$ = 3 for the whole graph. Thus, it seems likely that these instances can be quickly solved by the algorithm from Theorem 3, running in $O\left(2^{{q^{\prime }}_{v}}\cdot n^{2}\cdot q_{v}^{\prime }\right)$ time. One possible explanation for the low value for these parameters is that the networks exhibit a linear structure. For example, if each protein can be assigned a distance to the nucleus, and interactions mostly transport information to or from the nucleus, then we would expect to have only few cross pairs.

The parameter *m_v _*could be expected to be not too high in biological networks, since otherwise this would make the network less robust, since elimination of one vertex would disrupt too many paths. However, one vertex in the tree under consideration can actually correspond to a very large component in the original graph, which weakens this effect. Therefore, this parameter is more useful in sparser graphs, where not too many graph vertices are joined into a tree vertex. However, for the given instances, it seems small enough to be exploited only for fairly small instances, where other parameters would give good results, too.

The parameter *m_e _*could similarly be expected to be low in sparse networks; however, the NP-hardness result already for *m_e _*≥ 3 (Theorem 5) makes practical use of this parameter unlikely.

## Conclusions

We started a parameterized complexity analysis of (WEIGHTED) MAXIMUM TREE ORIENTATION, obtaining a more fine-grained view on the computational complexity of this NP-hard problem. In this line, there are still several challenges for future investigations. For instance, it is open whether MTO is fixed-parameter tractable with respect to the parameter "number of satisfied pairs" (*n *- *k*). Further, in the spirit of "distance-from-triviality parameterization" [19,20] it would be interesting to study the parameterized complexity of MTO with respect to the parameter "number of all possible pairs minus the number of input pairs"--recall that for parameter value zero MTO is polynomial-time solvable [11]. MTO restricted to stars is still NP-hard, but then at least one quarter of all input pairs can always be satisfied [3]. Hence, it would be interesting to study above guarantee parameterization [15,20] with respect to the number of satisfied pairs. MTO can be translated into a vertex covering problem (see Proposition 1) on a graph class that is *K*_4_-free--this motivates to study whether vertex covering on this graph class can be done faster than on general graphs. Clearly, MTO brings along numerous further parameters and parameter combinations which can make a more comprehensive multivariate complexity analysis [20] very attractive. Often, it is desirable to not only list a single solution, but to enumerate all optimal solutions. Our dynamic-programming-based algorithms seem suitable for this. Following Gamzu et al. [8] and extending the studies for MTO as pursued here to the more general case of mixed graphs with partially already oriented edges is of high interest. First steps in this direction have very recently been undertaken by Silverbush et al. [9] and Elberfeld et al. [26]. Finally, it seems promising to examine the parameters based on cross pairs in other networks such as communication networks, and to try to exploit these parameters for other hard network problems.

## Competing interests

The authors declare that they have no competing interests.

## Authors' contributions

All authors contributed more or less equally, RN initiating the study of MTO under the viewpoint of multivariate complexity analysis and JU coming up with the major algorithmic ideas which have been worked out in more detail by DK. All authors read and approved the final manuscript.
